# Communicating a Positive Result at Newborn Screening and Parental Distress

**DOI:** 10.3390/ijns9030038

**Published:** 2023-07-14

**Authors:** Elisa Lastrucci, Marta Daniotti, Elena Procopio, Giusi Scaturro, Flavia Tubili, Rosanna Martin, Giancarlo la Marca

**Affiliations:** 1 Department of Experimental and Clinical Biomedical Sciences, University of Florence, 50019 Florence, Italy; dott.elisalastrucci@gmail.com; 2 Metabolic Diseases Unit, Neuroscience Department, Meyer Children’s Hospital IRCCS, 50139 Florence, Italy; marta.daniotti@meyer.it (M.D.); elena.procopio@meyer.it (E.P.); giusi.scaturro@meyer.it (G.S.); flavia.tubili@meyer.it (F.T.); 3 Psychology Unit, Meyer Children’s Hospital IRCCS, 50139 Florence, Italy; rosanna.martin@meyer.it; 4 Newborn Screening, Biochemistry and Pharmacology Laboratory, Meyer Children’s Hospital IRCCS, 50139 Florence, Italy

**Keywords:** positive result, newborn screening, familiar stress

## Abstract

The assumption of this study is strictly connected to the need to focus and to know more about the impact on the psychological state of the parents whose newborn babies get a positive result at Expanded Newborn Screening (ENS). As clinical experience shows us, this aspect seems to have a potentially lasting resonance on the way the disease will be managed and handled in the family, leading to potential negative effects and repercussions on the child’s wellbeing and on the quality of life within the family. On the basis of this and on the evidence emerging from a review of the literature, this study aims to investigate and objectify possible distress indicators elicited at the moment of the communication of a positive result at ENS. Questionnaires containing the Beck Depression Inventory-II, the State-Trait Anxiety Inventory-Y, and the Short Form 36 Health Survey tests were administered to the parents of 87 newborns who received positive results at ENS. The parents of 32 babies expressed the presence of discomfort potentially related to the communication of a positive result at ENS.

## 1. Introduction

### 1.1. The Metabolic Diseases and the Expanded Newborn Screening

The inherited metabolic disorders represent a class of hereditary diseases caused by the impaired functioning of a metabolic pathway. They always depend on a genetic defect (a DNA mutation) that causes the lack of the production of an enzyme or of its cofactor or their production in a non-functional way.

Early diagnosis is of high value for the prognosis, since (in treatable disorders) it allows prompt and specific treatment and can prevent clinical problems subsequent to the onset or worsening of the disease.

The Expanded Newborn Screening begins with a blood draw from the newborn baby’s heel in the first days of life (in Italy, for instance, between the forty-eighth and the seventy-second hour after birth). Through sophisticated laboratory methodologies, it is possible to identify, in a short period of time, biochemical changes that can represent a warning sign of the disease.

In the last decades, there has been a remarkable expansion of the screened diseases panel at birth, thanks to the introduction of particularly efficient laboratory techniques (tandem mass spectrometry) that allow the possibility to screen over 40 congenital metabolic diseases from a single droplet of blood. The aim of the program is to select among the whole neonatal population subjects who show biochemical changes that can be indicators of metabolic diseases, to carry out a diagnostic assessment, and, in case of a confirmed diagnosis, to establish an adequate treatment for each patient.

The Italian Expanded Newborn Screening Programme, which uses liquid chromatography–tandem mass spectrometry (LC-MS/MS), is mandatory by national law since 2016 [1]. The first project for ENS in Italy started in Tuscany in January 2002. Officially mandated by legislative action, the program has screened all babies born in Tuscany (now approximately 700,000) for selected aminoacidopathies, beta-oxidation fatty acid deficiencies, urea cycle defects, three lysosomal storage disorders (Pompe, Fabry and MPS I) (since 2014), severe combined immunodeficiencies (since 2013), spinal muscular atrophy (since 2019), in addition to congenital hypothyroidism, galactosemia, cystic fibrosis, and biotinidase deficiencies.

### 1.2. The Research on the Impact of a Positive Result at ENS on Families

The choice to set this study in the direction of measuring the impact of communicating a positive result at newborn screening was preceded by a review of literature written in the last fifteen years about the topic. The main emerging aspects were:

- The limited amount of past studies which tried to measure the parents’ distress upon receiving positive results and the potential consequences [2,3];
- Clarifying that the quantity, quality, and rhythm of the information process involving the parents is as fundamental as considering the timing of the communication of a positive result in a particular phase of the parents’ life. In fact, planning carefully the quantity, quality, and rhythm of the information process that will be provided to the parents (written support, understandable language, multi-language translation, communicator competence, etc.) seems to be fundamental in reducing the potential distress experienced by the parents [4];
- The consideration of the timing of the communication of a positive result at ENS in the parents’ life is also important: this communication occurs while the parents are already under the stress of managing a newborn, experiencing sleep deprivation, and for some, navigating the skills of being first-time parents. This delicate phase of a parent’s life can be complicated further not only by the communication of a positive result and the realization that their child may have an inherited disorder, but also by not having enough information about the screened diseases [4];
- The published studies about this topic are not only mainly focused on one specific disease, such as cystic fibrosis, phenylketonuria, congenital adrenal hyperplasia, or a specific result at ENS (ex. the “false-positive”), but the majority of them are also in the large part qualitative and retrospective and they usually only involve the mothers and not the fathers [5,6,7,8,9,10,11,12,13,14,15,16,17].

There are some lines of concordance in literature:

- Having an adequate knowledge about the NBS process seems to reduce the anxiety and distress of parents but few parents are educated about how the process work;
- There is a general and wide lack of knowledge among parents about the screened diseases;
- A criticism is represented by the lack of preparedness of personnel who will be in charge of communicating a positive result to parents after ENS;
- Factors that may influence a lower level of satisfaction from parents during the ENS process are a long wait between the phone call communicating a positive result and the retesting, parents who are young, and belonging to an ethnic minority and/or to a lower social and cultural standard than average;
- In past studies, the parents who receive a positive result at ENS usually express a positive opinion about the process itself, regardless of its final outcome;
- Social support seems to be very important in reducing parental distress;
- Many studies show how parents, especially after receiving the first phone call from the ENS center and before the retesting, usually start a tireless search for information on the internet, and this ends up being mostly useless and harmful to them [15];
- There is a lack of data about the way information is provided to parents; nevertheless, it seems that when information is provided by an expert and when parents wait less than three days between the first phone call and the retesting they usually express a lower level of distress [3].

There are also some lines of discrepancy in the examined literature: these emerge particularly from studies focused on the false-positive outcomes at ENS. Some studies highlight the persistence of negative psychological consequences after one year or more after ENS. For example, there is a general parental perception of increased physical vulnerability of the child such as more frequent hospitalizations and urgent care visits. On the other hand, other studies show that the perception of increased physical vulnerability of the child ceases to exist after the conclusion of the diagnostic assessment process [9,17].

## 2. Methods

### 2.1. Objectives

This study has been conducted by a team of metabolic specialists at IRCCS Meyer Children’s Hospital in Florence using many years of clinical practice. The need of providing completeness to the studies conducted in the field of ENS joined the concrete needs emerging from the interactions between doctors and parents getting the communication of a positive result at ENS, including, thus, the psychological dimension. In particular, the study’s goals are:

- Quantify possible indicators of distress that parents experience at the moment they receive communication of a positive result at ENS;
- Measure the amount of distress that parents experience in the exact moment they receive a positive result and not retrospectively;
- Involve both mothers and fathers;
- Investigate the impact of the communication of a positive result at ENS independently from the specificity of the suspected disease;
- Carry out a study that includes both true positive (TP) and false positive (FP) cases.

### 2.2. Subjects

This study involves parents whose newborn babies get a positive result at ENS and who are born in Tuscany and in Umbria, with the possible exclusion of those parents who have not mastered the Italian language enough to understand the questionnaires.

The Institutional Ethics Board of Meyer Hospital of Florence approved this study (No. 42/2014; 43/2020). The authors have complied with the World Medical Association Declaration of Helsinki regarding the ethical conduct of research involving human subjects.

### 2.3. Instruments

The following battery of tests has been chosen for the purpose of the study:

- The Beck Depression Inventory-II,
- The State-Trait Anxiety Inventory-Y
- The Short Form 36 Health Survey.

#### 2.3.1. The Beck Depression Inventory (BDI II)

The BDI was created by Aaron Beck in 1961 and there were three editions of it [18,19,20,21,22,23]. BDI-II (ed. 1996) is the most currently used. It consists of 21 multiple choice questions and is a self-report questionnaire used to measure the severity of symptoms in depressed subjects or the presence of depression in people who represent normal populations; it can also be used to monitor either the changes of the scores in time and the effectiveness of treatment.

Using the current edition, subjects are asked how they have been feeling for the last two weeks. The possible answer consists of four possibilities on a scale from 0 to 3; higher total scores indicate more severe depressive symptoms. The questionnaire returns a total score and can be classified into two domains:

- The somatic–affective domain, which refers to manifestations that can be a result of depression, such as loss of interest, fatigue, changes in sleep and appetite, tears, agitation, etc.
- The cognitive domain, which includes manifestations such as pessimism, sense of guilt, self-criticism, low self-esteem, etc.

A total score corresponding to a percentile that is in the range between the 85th and the 90th means a condition of dysphoria with psychopathological issues; a range between the 91st and the 95th indicates a situation of dysphoria involving great difficulty and discomfort whereas a percentile beyond the 95th indicates a situation of particular difficulty and in some cases severe depression [24,25,26,27,28].

#### 2.3.2. State-Trait Anxiety Inventory (STAI-Y)

The STAI-Y is an easy-to-use tool used to detect and measure anxiety. It was created by Spielberger, Gorush, and Lushene in 1983 [29,30,31]. It is a self-report questionnaire on a “Likert scale”: the subject estimates how representative several statements are regarding their behavior on a scale from 1 to 4 (1 = not at all, 4 = very much). The questions are grouped into two scales:

- The state anxiety scale indicates how anxious the subject is feeling “in that very moment” and this is connected to a stimulus situation, so it is transient and of varying intensity;
- The trait anxiety scale refers to a personal tendency to perceive situations as stressful, dangerous, and threatening and to react with particular intensity; therefore, it refers to a longer-lasting and more stable personality condition that exists independently from a particular or specific situation.

The scores will normally be given in a range from 20 to 80; the higher the score, the more intense the anxiety. The STAI-Y can be useful to obtain a generic index of stress since it does not refer to specific situations. A cut-off value of 40 or above can be predictive of anxious symptomatology. According to a scalar criterion, it is possible to define the level of severity: values between 40 and 50 indicate mild symptoms, between 50 and 60 moderate symptoms, and >60 severe symptoms; so, a total score corresponding to a percentile beyond the 85th indicates the presence of severe anxiety [32,33,34].

#### 2.3.3. The SF-36 (Short-Form36 Items Health Survey)

This questionnaire is made up of 36 questions with the aim of assessing the quality of life. It is not specific for pathology, age, and/or treatment and it assesses the subjective perception of eight possible health dimensions connected to different levels of activity and wellbeing. It was developed by Ware and Sherbourne [35,36,37,38,39].

The uses of this questionnaire are usually aimed toward the assessment of the individual state of health, the evaluation of the costs for treatment, and the comparison and monitoring of the burden of having different diseases (this last dimension is closer to the interest that led to this study).

The SF-36′s questions are divided into 8 health domains:

1. Physical Functioning (10 items);
2. Role limitations due to physical health (4 items);
3. Role limitations due to emotional state (3 items);
4. Fatigue (4 items);
5. Emotional Wellbeing (5 items);
6. Social Activities (2 items);
7. Pain (2 items);
8. General Health perception (5 items).

The scores are calibrated so that 50 can be considered the average. Every scale is transformed into a scale from 0 to 100 assuming that every question is represented by the same weight. A score of 0 is equivalent to the maximum disability and a score of 100 to the absence of it. We considered with particular interest the scores under 50, in order to assess negative perceptions of health within the eight health domains.

### 2.4. The Procedure

In Italy, the ENS is mandatory by national law; therefore, written consent is not required, with the exception of some specific regional research pilot projects.

Comprehensive informative sheets (translated into the most diffuse spoken language in the region) are given and explained to the family in the first 48 h of life after the partum by a pediatrician or a gynecologist. The informative material explains the following: what ENS is; the aim of ENS; how it is carried out; what diseases are detected by ENS; and when the parents will know the results. Moreover, it has been reported that when the newborn tests positive, he/she will be called back to the birth center or reference metabolic unit for further investigations.

In our study, when the screening procedures (first, if available second tier test and in low-risk disorders retesting on a second DBS) have been completed and a positive result has been found, the newborn is rescheduled for a “first visit” by a metabolic doctor at IRCCS Meyer Children’s Hospital with a phone call. Usually, the psychologist is introduced by the metabolic physician, so that at the end of the visit, the newborn parents can be given psychological support if requested. Afterward, the study and its objective are introduced to the parents with a short explanation of the questionnaires. The study requires that the questionnaires are filled in and given back within the day of the appointment. Socio-demographic data, the specific biochemical alteration highlighted by the ENS, and information about the suspected and final diagnosis are written down for each proband. The communication process was identical for all parents, in order to guarantee the best level of standardization. Parents were also offered time to discuss and ask about eventual doubts, resistances, or concerns regarding the questionnaires and their completion. 

## 3. Results

### 3.1. Socio-Demographic Variables

The questionnaires were given to parents of 87 babies born in Tuscany and in Umbria who received a positive result at ENS over a period of time starting in May 2019 through February 2022. Parents of 32 babies (34.5%) completed and gave back the questionnaires: 31 fathers (51.7%) and 29 mothers (48.3%). In summary, both parents of 28 babies, the fathers of 3 babies, and the mother of 1 baby completed the questionnaires. Nine foreign parents (15.0%) (Albania, Romania, Nigeria) with a good knowledge of the Italian language completed the questionnaires. There were no mixed-race couples. 

Twenty-nine fathers (93.6%) had a job at the time of the NBS and two (6.4%) were unemployed (both not Italian). Twenty-one mothers (72.4%) had a job and eight (27.6%) were unemployed (four Italian, four non-Italian). Overall, 83.3% of the parents had a job at the time of NBS, and 16.7% were unemployed (the majority were mothers).

The positive results at ENS include the following: 2 cases of suspected Pompe disease; 10 cases of hyperphenylaninemia; 5 cases of suspected beta-oxidation deficiency (2 MCAD; 2 VLCAD; 1 SCAD); 3 cases of suspected methylmalonic acidemia (MMA); 2 cases of suspected mucopolysaccharidosis I (MPS1); 5 cases of suspected Fabry disease; 1 case of suspected galactosemia, 2 cases of hypermethioninemia, and 2 cases of carnitine deficiency.

### 3.2. The Beck Depression Inventory (BDI-II)

Fifteen parents (25.1%), of which seven fathers (22.6%) and eight mothers (27.6%) received a total score that could indicate a dysphoria condition with pathological aspects. Considering the somatic–affective area, 19 parents (31.7%), of which 10 fathers (32.4%) and 9 mothers (31.0%), showed scores that could be related to the presence of clinically significant symptoms. Finally, 12 parents (20.0%) received scores in the cognitive area, which indicate discomfort and dysphoria, of which were 6 fathers (19.3%) and 6 mothers (20.7%).

### 3.3. The State-Trait Anxiety Inventory (STAI-Y)

In regards to the state anxiety scale, 51 parents (85.0%) showed scores indicating the presence of anxiety in a range from mild to severe, of which were 24 fathers (77.4%) and 27 mothers (93.1%). In addition, 21 parents (35.0%) received scores indicating severe state anxiety, of which were 9 fathers (29.0%) and 12 mothers (41.4%). As far as trait anxiety is concerned, six parents (10.0%) presented scores that indicate a severe condition of anxiety, of which were three fathers (9.7%) and three mothers (10.3%).

### 3.4. The SF-36

The domain connected to the worst perception by parents is the “fatigue”: 21 parents (35.2%), 10 fathers (32.3%), and 11 mothers (37.9%) presented scores under the average of 50, thus, indicating a negative functioning. Seventeen parents (28.3%) showed scores that can be related to a negatively perceived functioning as far as the “role limitation due to the emotional state” is concerned: seven fathers (22.6%) and ten mothers (34.5%); seventeen parents as well showed scores expressing a negatively perceived functioning about the “social activity” domain, of which were nine fathers (29%) and eight mothers (27.6%). Sixteen parents (26.7%) received scores relatable to a negative perception of health in the “role limitation due to physical state” domain; among these were seven fathers (22.6%) and nine mothers (31.0%). Fifteen parents (25.0%) showed an intense perception of “pain” with a clear predominance of mothers: eleven mothers (37.9%) and four fathers (12.9%). A minor impairment in quality of life was highlighted in the “physical functioning” and “general health” domains; in both domains, four parents (6.7%), respectively, two fathers (6.4%) and two mothers (6.4%), and only three fathers (9.7%) and one mother (3.4%) presented a negative perception of quality of life.

The overall scores indicating the presence of discomfort for the parents have been reported in Table 1.

## 4. Discussion

The aim of this study was to measure the stress experienced by parents in the moment of the communication of a positive result at ENS. This measurement was made by identifying three potential indicators: the presence of depressive symptoms, anxiety, and the quality of life perceived by the parents. As far as the BDI-II scores are concerned, particular attention was given to the three main categories of scores: the total score, the somatic–affective score, and the cognitive score. Attention was also given to the percentage of parents (general sample, fathers sample, and mothers sample) who presented clinically significant scores (above the 85th percentile) that could, thus, indicate the presence of a condition of dysphoria with pathological aspects in a range of intensity from “mild” to “severe”. The Beck Depression Inventory highlighted that, in our sample, the area where most parents (and the highest percentage of them) received scores indicating the presence of dysphoria was in the “somatic–affective area” (indicated by the loss of interests, loss of energy, changes in sleep and appetite, and to the presence of agitation and tears, etc.). We also highlighted that those changes are more prominent in mothers than in fathers in the same domain.

The STAI questionnaire results showed a large difference in the anxiety-state scores between mothers (higher/worse) and fathers, whereas the difference between their scores on the anxiety-trait scale was much smaller. However, the comparison between the two scales of anxiety seemed to confirm a form of anxiety, particularly intense in mothers, that can potentially be related to the communication of a positive result at ENS.

By analyzing the SF36 results, we considered those scores (and the relative percentages of parents) that could indicate a negative health perception in the eight health domains. The SF36 showed that those parents who received scores indicating the presence of discomfort are a minority when compared to those parents who received scores relatable with positive functioning, as well as for what emerged from the other two questionnaires. It is interesting to notice that a higher percentage of parents referred to the presence of discomfort in the “somatic” area (the “energy” domain in the SF36 and “somatic–affective” domain in the BDI-II). On the other hand, it is not surprising to assume that parents who received a positive result at ENS could show the presence of discomfort on a somatic level rather than on a cognitive level. Clearly, in order to better investigate this aspect further studies including the presence of a control sample (parents whose baby did not get a positive result at ENS) are needed. Furthermore, we could not help noticing an important difference in the scores between fathers and mothers in the “pain” domain, but we could attribute this to the delivery, considering its temporal proximity to the moment of the compilation of the questionnaires, therefore, influencing the difference in scores between mothers and fathers as far as the “role limitations due to physical state” is concerned.

It is very interesting that, even though this group is relatively small in our sample, non-Italian and unemployed parents received scores indicating a severe state of discomfort in all three questionnaires, without exception; specifically, six parents (10.0%), of which were four mothers (13.8%) and two fathers (6.4%). This could confirm the observations in literature that highlight the fact that belonging to a cultural minority and being unemployed could contribute, among other factors, to a more severe reaction to the ENS process caused by the intense non-related stress experienced at the moment of the communication of a positive result at ENS [3,40].

The last important point to discuss is the “timing” of the administration of the questionnaires which could possibly have some bias. Our objective was to measure the overall discomfort during the first day-hospital visit immediately after the in-person communication of positivity at the metabolic center. The choice of the timing was to measure the stress experienced by parents in the ‘heat of the moment’. After observing the reaction of parents immediately after the communication of a positive test result, we concluded that testing their distress was not appropriate in that moment. Instead, parents often showed the need to focus mostly on understanding what was going on, what was wrong with their babies, what they had to do, how to handle what was happening, and how to get a sense of orientation rather than getting in touch with their emotions or expressing them. Therefore, parents may be non-accessible regarding their emotive state, resulting in resistance to filling out the questionnaires. The literature seems to confirm that the parents’ needs in the moment of the communication of a positive result at ENS are mostly related to information and understanding the pragmatic aspects of the disease [15,41,42,43].

## 5. Conclusions

The absence of a control sample, as well as the small size of our test, limits the generalizability of the study findings. As a matter of fact, this study does not allow us to measure “the real impact” of the communication of a positive result at ENS due to the limited factors that were analyzed. A further area to be explored is evaluating the parent’s reaction to a communication of a positive result of “suspected diseases”. The small size of our sample population prevented the study from researching possible differences in this direction from emerging. Other future areas to be explored could be the investigation of those characteristics and qualities of parents that could constitute intervenient variables influencing and modulating the relationship between the event and its impact.

This knowledge could help us on a pragmatic level with the aim of preventing or minimizing, as much as possible, the negative effects regarding the parents’ psychological state and quality of life after receiving an ENS-positive result.

The research on the impact of parents’ stress upon receiving the communication of a positive result at NBS is still sparse. It is necessary to expand this field, starting from the preliminary aspects that could constitute the foundations for further studies.

## Figures and Tables

**Table 1:** BDI-II, STAI-Y, and SF-36 scores indicating discomfort in parents.

	Parents	Fathers	Mothers
BDI: somatic–affective area	19 (31.7%)	10 (32.4%)	9 (31.0%)
BDI: total	15 (25.1%)	7 (22.6%)	8 (27.6%)
BDI: cognitive area	12 (20.0%)	6 (19.3%)	6 (20.7%)
STAI: state	21 (35.2%)	9 (29.0%)	12 (41.4%)
STAI: trait	6 (10.0%)	3 (9.7%)	3 (10.3%)
SF36: fatigue	21 (35.2%)	10 (32.3%)	11 (37.9%)
SF36: social activity	17 (28.3%)	9 (29.0%)	8 (27.6%)
SF36: role limitations due to emotional state	17 (28.5%)	7 (22.6%)	10 (34.5%)
SF36: role limitations due to physical state	16 (26.8%)	7 (22.6%)	9 (31.0%)
SF36: pain	15 (25.4%)	4 (12.9%)	11 (37.9%)
SF36: emotional wellbeing	6 (9.9%)	4 (12.9%)	2 (6.9%)
SF36: physical functioning	4 (6.4%)	2 (6.4%)	2 (6.4%)
SF36: general health	4 (6.7%)	3 (9.7%)	1 (3.5%)

## Data Availability

Data from single questionnaire is unavailable due to privacy.
